# Tissue specific expression of Myosin IC Isoforms

**DOI:** 10.1186/1471-2121-15-8

**Published:** 2014-03-11

**Authors:** Neil L Sielski, Ivanna Ihnatovych, Jacob J Hagen, Wilma A Hofmann

**Affiliations:** 1 Department of Physiology and Biophysics, University at Buffalo-State University of New York, 3435 Main Street, Buffalo, NY 14214, USA

**Keywords:** Myosin IC, Isoforms, Gene expression, Tissue specificity, Protein expression

## Abstract

**Background:**

Myosin IC is a single headed member of the myosin superfamily that localizes to the cytoplasm and the nucleus and is implicated in a variety of processes in both compartments. We recently identified a novel isoform of myosin IC and showed that the *MYOIC* gene in mammalian cells encodes three isoforms (isoforms A, B, and C) that differ only in the addition of short isoform-specific N-terminal peptides. The expression pattern of the isoforms and the mechanisms of expression regulation remain unknown.

**Results:**

To determine the expression patterns of myosin IC isoforms, we performed a comprehensive expression analysis of the two myosin IC isoforms (isoform A and B) that contain isoform-specific sequences. By immunoblotting with isoform-specific antibodies and by qRT-PCR with isoform-specific primer we demonstrate that myosin IC isoforms A and B have distinct expression patterns in mouse tissues. Specifically, we show that myosin IC isoform A is expressed in a tissue specific pattern, while myosin IC isoform B is ubiquitously expressed at comparable levels in mouse tissues.

**Conclusions:**

The differences in the expression profile of the myosin IC isoforms indicate a tissue-specific *MYOIC* gene regulation and further suggest that the myosin IC isoforms, despite their high sequence homology, might have tissue-specific and isoform-specific functions.

## Background

Myosin IC (formerly known as myosin I-β; [1]) is a single headed class I myosin that localizes to the nucleus and the cytoplasm. In the cytoplasm, myosin IC has been implicated, among other processes, in lipid raft arrangements [2], transport of vesicles containing membrane proteins such as the glucose transporter [3], and in ion channel regulation in hair cells of the inner ear [4-6]. In the nucleus, myosin IC is involved in various aspects of transcription [7-10], in chromatin remodeling [11-13], and in dynamic organization of chromosomal structures [14].

**Figure 1:**
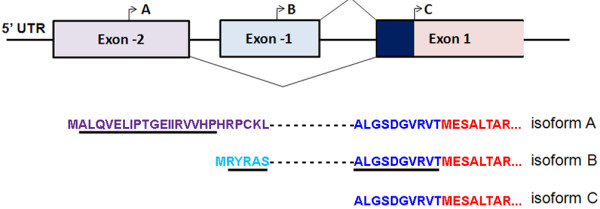
**Schematic of myosin IC isoform-specific sequences and recognition site of antibodies.** The upper panel depicts the 5’ region of the mammalian myosin IC gene including the exons that code for isoform-specific N-terminal peptides and the transcription initiation sites for the isoforms. The lower panel shows the N-terminal amino acid sequences of the isoforms. Underlined are the peptide sequences that were used as immunogen to create isoform-specific antibodies [9,18].

Initially, it was thought that the cytoplasmic and nuclear functions of myosin IC are facilitated by two isoforms that are encoded by the *MYOIC* gene, known as myosin IC and nuclear myosin I (NMI) [9,15]. However, a number of recent studies showed that both isoforms can localize to the cytoplasm and the nucleus [16,17]. In addition, we recently identified a previously unknown isoform of myosin IC and demonstrated that the *MYOIC* gene in mammalian cells encodes three isoforms: isoform A (newly discovered [18]), B (formerly NMI [9,15]), and C (formerly known as myosin IC [19]). As shown in Figure 1, the only difference between the three isoforms are additional, short N-terminal peptide sequences of 35 and 16 amino acids that are added to isoforms A and B respectively that are derived from upstream exons [18].

Interestingly, despite the high sequence homology, initial studies on isoform localization and function indicate that the myosin IC isoforms localize to different cellular compartments and are functionally distinct [17,18]. However, the underlying factors that facilitate the functional difference between the isoforms are not fully understood. In addition to the potential functional differences between the isoforms and their distinct intracellular localizations, our previous analysis of expression of the newly identified myosin IC isoform A in tissue culture cells also indicated a potential difference in expression patterns between the isoforms [18].

Previous studies analyzing expression of total myosin IC with antibodies directed against an epitope in the C-terminal domain that is common to all myosins as well as studies analyzing protein and mRNA expression of myosin IC isoform B (NMI) in a variety of organisms and tissues demonstrated a ubiquitous and conserved expression of myosin IC [20-22]. However, our comparison of myosin IC isoforms A and B expression in HeLa, COS-7, and NIH 3T3 cells showed that while all three cell types express myosin IC isoform B at comparable levels, isoform A was strongly expressed only in COS-7 cells but could barely be detected in NIH 3T3 and HeLa cells [18] which suggests a difference in the expression pattern of the myosin IC isoforms. Therefore, we extended our studies and present here a comprehensive analysis of the expression pattern of myosin IC isoform A and B in mouse organs and tissues.

## Results and discussion

As shown in Figure 1, only two of the three myosin IC isoforms that are expressed by the *MYOIC* gene, namely isoforms A and B, contain nucleotide and amino acid sequences that are isoform-specific and thus can be detected individually [18]. To determine protein expression of the two isoforms, we performed immunoblot analysis of a panel of 33 different organs and tissues that were collected from 2-4 month old male and female C57Bl/6 mice. Protein extracts were analyzed using antibodies that recognize the individual isoforms. Figure 1 shows a schematic of the 5’ region of *MYOIC*, the resulting N-terminal amino acid sequences of the myosin IC isoforms, and the amino acid sequences that are recognized by the isoform-specific antibodies. The antibody that recognizes specifically myosin IC isoform A, is a monoclonal antibody that was generated using a peptide sequence that is encoded by the isoform A-specific exon -2 as immunogen [18]. The antibody that recognizes myosin IC isoform B (NMI) is a polyclonal antibody that was generated using the isoform B-specific 16 amino acid long N-terminal peptide as immunogen [9].

**Figure 2:**
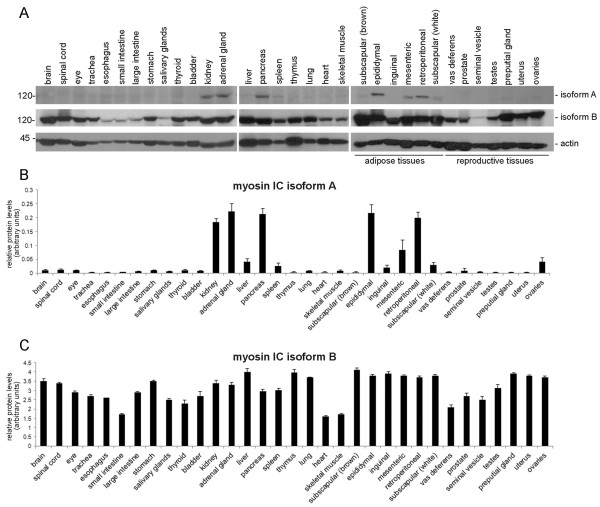
**Protein expression of myosin IC isoforms A and B in mouse tissues.** Detection of myosin IC isoforms A and B in various mouse tissues by immunoblot analysis using antibodies specific to myosin IC isoforms A, isoform B, and actin (control). **A)** Representative immunoblots of mouse tissue extracts that were analyzed using the indicated antibodies. Relevant molecular weight markers are indicated on the left. **B)** Histogram presenting the average densitometric intensity of isoform A expression normalized to actin. **C)** Histogram presenting the average densitometric intensity of isoform B expression normalized to actin. Results are presented as means + standard deviation; n = 3-6 (depending on tissue type).

Probing the organ and tissue extracts with the myosin IC isoform A-specific antibody (Figure 2A and B) showed that isoform A is expressed in a tissue-specific pattern. Expression levels are high in kidney, adrenal gland, pancreas, and in a subset of adipose tissues, specifically in tissue from epididymal and retroperitoneal adipose depots. In addition, moderate levels of isoform A protein can be consistently detected with only slight variations, in protein extracts from liver, spleen, ovaries, and tissue from mesenteric, inguinal, and white subscapular adipose depots. In the remaining 22 analyzed organs and tissues isoform A protein is only present at minimal, barely detectable levels.

In contrast to the tissue-specific expression of isoform A, myosin IC isoform B protein is easily detectable in all analyzed tissues and shows only moderate variations in expression levels, with the lowest expression observed in skeletal muscle and heart and the highest expression observed in liver, thymus, and in the various adipose depot tissues (Figure 2A and C). However, it should be noted that the maximum difference in relative expression of isoform B between the analyzed tissues is only 2.3 fold while the difference in relative expression of isoform A between the various tissues is up to 22 fold (compare Figure 2B and C).

**Figure 3:**
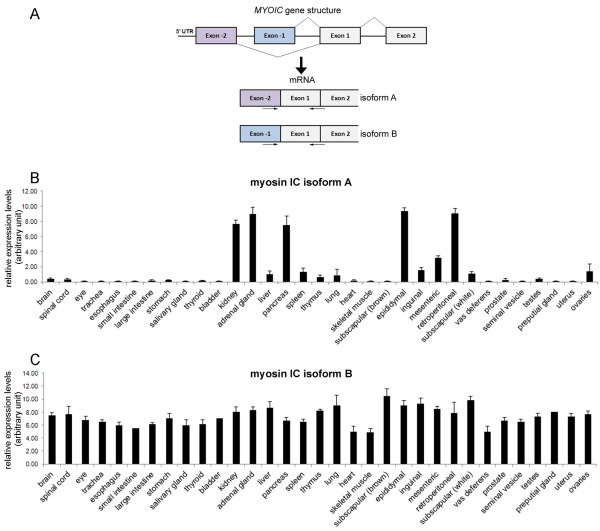
**mRNA expression of myosin IC isoforms A and B in mouse tissues. A)** Schematic depicting the 5’ region of the MYOIC gene and the resulting mRNA structure. The target sequence region and location of primer used for qRT-PCR to detect myosin IC isoforms A and B are indicated by arrows. **B)** Quantitative real-time PCR analysis of mRNAs expression levels of myosin IC isoform A normalized to GAPDH. **C)** Quantitative real-time PCR analysis of mRNAs expression levels of myosin IC isoform B normalized to GAPDH. Results are presented as means *+* standard deviation; n =3-6 (depending on tissue type).

We next analyzed if this difference in expression patterns between myosin IC isoforms A and B is also observed on mRNA level. We measured mRNA expression of the isoforms by quantitative real time PCR (qRT-PCR) using isoform-specific forward primer and reverse primer that bind to a region common to both isoforms. The location of the primer is shown in Figure 3A. We found that the mRNA expression profiles of myosin IC isoforms A and B correlate to the protein expression profiles indicating that regulation of expression takes place on gene level. Comparable to the protein data, mRNA expression of myosin IC isoform A is high in kidney, adrenal gland, pancreas, and in adipose tissue from epididymal and retroperitoneal depots. In addition, moderate levels of isoform A mRNA are expressed in liver, spleen, ovaries, and adipose tissue from mesenteric, inguinal, and subscapular white fat depots. In the remaining 22 analyzed organs and tissues isoform A mRNA can barely be detected (Figure 3B).

In contrast, isoform B mRNA is expressed in all analyzed tissues with only slight variations in expression levels (Figure 3C).

In summary, we have identified and characterized a tissue-specific expression pattern of a recently identified, novel myosin IC isoform and we demonstrate that two of the myosin IC isoforms exhibit substantial differences in the expression profiles. Our data show that in contrast to the ubiquitously expressed myosin IC isoform B, the newly discovered myosin IC isoform A exhibits tissue-specific expression patterns on both, protein and mRNA levels. Previous studies analyzing the cellular distribution of the myosin IC isoforms in COS-7 cells, i.e. in a cell line that expresses all three myosin IC isoforms at high levels [18], revealed that each of the three isoforms localizes to specific cellular regions and interacts with different binding partners [17,18] which strongly suggests isoform-specific cellular functions. Our identification of differential expression profiles further strengthens the notion that the myosin IC isoforms are functionally distinct. Future work is aimed at understanding the regulatory mechanisms that lead to tissue-specific expression of myosin IC isoform A and at identifying potential tissue-specific functions of this isoform.

## Conclusions

The *MYOIC* gene expresses three different isoforms two of which exhibit significant differences in expression patterns. While myosin IC isoform B is ubiquitously expressed, myosin IC isoform A exhibits a tissue-specific expressed pattern that suggests tissue-specific functions of this myosin IC isoform.

## Methods

### Antibodies

Figure 1 shows the isoform-specific sequences that were used to generate myosin IC isoform specific antibodies. Antibodies that recognize various isoforms of myosin IC are: 1. the anti-NMI antibody is a rabbit polyclonal antibody that was raised against the 16 amino acid long N-terminal peptide of NMI, here called isoform B [9,23] (Sigma-Aldrich, St Louis, MO); 2. the myosin IC-isoform A antibody is a mouse monoclonal antibody that was raised against the myosin IC isoform A specific N-terminal peptide and recognizes exclusively myosin IC isoform A [18]. Monoclonal antibodies to β-actin were obtained from Sigma (Sigma-Aldrich, St Louis, MO). Peroxidase-conjugated secondary anti–mouse or anti–rabbit antibodies were obtained from Jackson ImmunoResearch Laboratories (West Grove, PA).

### Animals and tissue collection

Tissue was isolated from male and female C57BL/6 mice 2–4 month old. All animal work was approved by the institutional animal care and use committee (University at Buffalo; protocol # PGY58128N). For each tissue type, tissues were extracted and analyzed from at least 3 male and 3 female mice.

### Immunoblot analysis

For preparation of protein extracts, tissues were homogenized by sonication at 4°C for 5–20 seconds (depending on tissue) in extraction buffer [1x PBS, 1% NP40; 5 mM EDTA; 2% SDS, 1% Na-deoxycholate, with protease inhibitors (Sigma-Aldrich, St Louis, MO)] followed by a 30 min incubation on a rocking platform at 4°C. The samples were then centrifuged at maximum speed in a tabletop centrifuge for 10 min at room temperature. The supernatant was used to determine protein concentration using a Bradford protein assay according to manufacturer’s instructions (Bio-Rad, Hercules, CA). Equal amounts of protein extracts (40 μg) were separated by 10% SDS-PAGE (sodium dodecyl sulfate-polyacrylamide gel electrophoresis) and transferred onto nitrocellulose membrane. After transfer, the membrane was cut followed by incubation of the lower half with antibodies specific to actin while the upper half was incubated with antibodies specific to myosin IC isoform A. Immunoreactive bands were detected by enhanced chemiluminescence. After detection of isoform A, the blots were stripped and re-probed with the antibody specific to myosin IC isoform B. Densitometric analysis was performed on the selected bands based on their relative intensities using ImageJ software.

### Quantitative real-time PCR (qRT-PCR)

Total RNA was isolated from mouse tissues using Trizol® reagent (Invitrogen, Carlsbad, CA) following the manufacturer’s instructions. 1 μg total RNA from each tissue sample was used to reverse transcribe into cDNA using Superscript III reverse transcriptase and oligo dT primer according to manufacturer’s instructions (Invitrogen, Carlsbad, CA). qRT-PCR was performed using the iCycler iQ Real-Time PCR Detection System (Bio-Rad, Hercules, CA) and primer that are indicated below that recognize specifically myosin IC isoform A (*homo sapiens:* NM_033375.4; *mus musculus*: XM_006532429.1) and myosin IC isoform B (*homo sapiens:* NM_001080950.1; *mus musculus:* NM_001080775). For quantification, triplicates were normalized to the average of the GAPDH housekeeping gene. For verification of sequences, the qRT-PCR product were separated on a 2% agarose gel, isolated from the gel and sent for DNA sequencing. Table 1 shows the primer used in qRT-PCR to detect isoform-specific expression levels of myosin IC isoforms A and B (see also schematic in Figure 3A).

**Table 1:** Primer used in qRT-PCR analysis

**Gene**	**Forward primer (5’-3’)**	**Reverse primer (5’-3’)**
*MYOIC* (isoform A)	ggagagatcatccgtgtggt	ggaccgatgtaggtataaatgagg
*MYOIC* (isoform B)	gcgctaccgggcatcg	ggaccgatgtaggtataaatgagg
*GAPDH*	ggtgaaggtcggtgtgaacg	ctcgctcctggaagatggtg

## Abbreviations

RT-PCR: reverse transcription polymerase chain reaction; qRT-PCR: quantitative real time polymerase chain reaction; SDS-PAGE: sodium dodecyl sulfate-polyacrylamide gel electrophoresis; PBS: phosphate buffered saline; 5’ UTR: 5’ untranslated region.

## Competing interests

The authors declare that they have no competing interests.

## Authors’ contributions

NLS, JJH, and II performed the immunoblotting experiments. NLS and JJH performed the qRT-PCR experiments. II and WAH conceived the study, participated in its design and interpretation, as well as drafting the manuscript. All authors read and approved the final manuscript.
